# Supplementary material for: Content-rich biological network constructed by mining PubMed abstracts
Source: BMC Bioinformatics. 2004 Oct 8;5:147. doi: 10.1186/1471-2105-5-147 (PMC528731; doi:10.1186/1471-2105-5-147)
Supplement: Additional File 5 — The original Chilibot query results of the term "long-term potentiation (LTP)" and 22 other terms, limiting the latest references analyzed to the years 1990, 1995, 2000, and 2004. [file 1471-2105-5-147-S5.bz2 › chilibotAdditionalFile5/ltp2000/html/ARC.html]

 


**ARC** (Input: ARC ) 

---


**Google Searches:** Entire Web  | EDU domain only  | PDF files only

.

**External Links:** OMIM |
LocusLink
| Swissprot
| GeneCards

  
**Maps of ARC**

Simple
Complete
graph in
radiant
tree
square
layout.

**New Hypothesis !**

**Synonyms** 

- arc   [PubMed]

**Synopsis**

- Moreover, we show that polysomal association of alpha CaMKII mRNA,   J Neurosci, 2000 
  [23]

- The data suggests that activation of the Bezold Jarisch reflex by 5 HT involves a glutamatergic synapse presumably located within the brainstem vagal reflex **arc**.  Neuropharmacology, 1987 
  [23]

- The present results suggest that **Arc** may play an important role in the neuronal plasticity through NMDA activation following focal cerebral ischemia.  Neuroreport, 1999 
  [23]

- Our results are consistent with the hypothesis that developmental neurotoxicity of **ARC** is due, in part, to the presence of ortho substituted PCB congeners.  Toxicol Lett, 1998 
  [21]

- None of the numerous neuroactive chemicals found in the NTS had met strict criteria as a neurotransmitter in the baroreflex **arc** until data suggested that the excitatory amino acid L glutamate GLU might be released from baroreceptor afferent terminals in the NTS.  Braz J Med Biol Res, 1997 
  [20]

- Therefore, it is suggested that the insulin induced coclustering of the EGF receptor with phospholipase Cgamma1 at the actin **arc** may allow for greater efficiency of signal transduction, resulting in the synergy observed for these two hormones in stimulation of DNA synthesis.  Immunol Cell Biol, 2000 
  [20]

- Only 5% of the control cells but 30 and 62% of the T3 treated 5 and 100 nM **ARC** showed this restricted myofibrillar phenotype.  J Clin Invest, 1996 
  [19]

- Thus, **Arc** appears to play a fundamental role in the stabilization of activity dependent hippocampal plasticity.  J Neurosci, 2000 
  [19]

- Next, we observed the formation of **arc** like bundles at the edges of contacting cells, a position suggestive of a role in lateral expansion of the contact.  Cell Motil Cytoskeleton, 1999 
  [19]

- Comparison of these results with those in the cat suggests the presence of a basic horizontal vestibulo ocular reflex that is very similarly organized, and corroborates the hypothesis that major behavioural differences in the performance of compensatory eye movements between species result from the properties of supplementary networks and NOT from differences in a common three neuron vestibulo ocular **arc**.  Eur J Neurosci, 1993 
  [17]

- We suggest that in developing embryos the neuralizing signal emanating from the organiser at first induces eIF4AII and the prospective neural crest in an **arc** low on the dorsal aspect of the embryo.  Development, 1997 
  [16]

- All EGF induced responses were abolished by the specific tyrosine kinase inhibitor pp60v **arc** 137 157, suggesting that EGF receptor tyrosine kinase activity is essential for G i 1 2 mediated activation of PLC gamma 1.  Am J Physiol, 1997 
  [15]

- AMPA receptor GluR1 immunoreactive levels in the magnocellular preoptic area mPOA, the arcuate nucleus **ARC**, and the suprachiasmatic nucleus SCN were found to be markedly elevated during the time of the LH surge in estradiol progesterone treated castrate rats compared to those of the vehicle only treated castrate rat.  Front Neuroendocrinol, 1994 
  [15]

- These results indicate that mu receptors may be autoreceptors on **ARC** beta endorphin neurons.  Neuroendocrinology, 1990 
  [14]

- **ARC** and PAG stimulated inhibited a nociceptive reflex tail dip in 52.5 degrees C water, and naloxone did NOT reliably reverse this inhibition.  Brain Res, 1995 
  [10]
